# Hostages to Social Networks and Mental Health

**DOI:** 10.1590/1516-3180.2025.1432.13122024

**Published:** 2025-03-31

**Authors:** Carmita Helena Najjar Abdo, Paulo Manuel Pêgo-Fernandes

**Affiliations:** I Associate Professor, Department of Psychiatry, Medicine School, Universidade de São Paulo (USP), São Paulo (SP), Brazil; Coordinator of the Program of Studies in Sexuality (ProSex) of the FMUSP, São Paulo (SP), Brazil; President, Brazilian Psychiatric Association (2017–2019), Rio de Janeiro (RJ), Brazil.; II Vice Dean, Faculdade de Medicina, Universidade de São Paulo (USP), São Paulo (SP), Brazil; Full Professor, Department of Cardiopneumology, Faculdade de Medicina, Universidade de São Paulo (USP), São Paulo (SP), Brazil; Director of the Scientific Department, Associação Paulista de Medicina (APM), São Paulo (SP), Brazil.

The Oxford dictionary's word of the year for 2024, "brain rot," refers to the perceived deterioration of mental or intellectual abilities caused by excessive consumption of material—especially online content—that is trivial or not very challenging. The emblematic choice of this word draws attention to a disastrous reality that affects a large portion of the world's population, posing serious risks to mental health and emotional balance. However, this expression is not new; it dates to 1854, when Thoreau, in his book *Walden*, criticized the tendency to devalue complex ideas—those open to multiple interpretations—in favor of simple ones, indicating intellectual decline.^1^

In recent decades, social networks usage among adolescents and young adults has risen dramatically, rising from 12% in 2005 to 90% by 2015.^2^ During the COVID-19 pandemic, this trend further intensified as people turned to social media as a coping mechanism during lockdown. By 2022, of the world's 7.91 billion people, 4.62 billion were active social networks users, spending an average of 6 hours and 58 minutes online daily, with 2 hours and 27 minutes specifically on social networking platforms.^3^

Brazil ranks second globally in terms of screen time, with Brazilians spending 56% of their day online—second only to South Africans, who spend 58.2% of their day on digital activities. Among the nine hours that Brazilian Internet spend **online each day**, four are dedicated to platforms such as Instagram, Facebook, TikTok, and X.^4^

The increasing use of social networks often leads to non-adaptive behaviors, collectively referred to in scientific literature as problematic use of social networks (PUSN), which manifests itself through various cyber behaviors, including:

- *Internet gaming disorder*: Recognized as a mental illness by the World Health Organization and included in the 11^th^ International Classification of Diseases (ICD-11).^5^ Symptoms includes prioritizing electronic games over other activities and losing control over their use, whether on a cell phone, computer, or video game.
- *FoMO ("Fear of Missing Out")*: Characterized by the anxiety stemming from the possibility of not participating in rewarding online experiences, leading individuals to feel compelled to constantly monitor their online social interactions.
- *Nomophobia ("No Mobile Phobia")*: Describes the anxiety experienced when being without a cell phone or disconnected from technology.
- *Phubbing* ("phone" + "snubbing"): Ignoring face-to-face social interaction in favor of using a cell phone.
- *Digital addiction, technology addiction, or digital dependence*: Involves excessive, compulsive use of electronic devices, negatively impacting daily activities.
- *Phantom text syndrome*: Reflects insecurity in virtual relationships, often characterized by distress when a response to a message is not immediate.
- *Phantom ringing*: The sensation of hearing or feeling ringtones and vibrations from a cell phone when the device is silent or absent.
- *Cyberchondria*: A compulsive tendency to search for symptoms and diseases online, often leading to exaggerated or irrational health concerns.
- *Selfitis*: An obsession with repeatedly taking selfies and sharing them on social networks, often reflecting a need for self-promotion.
- *Digital nausea*: Includes symptoms like disorientation, dizziness, and nausea caused by excessive interaction on social networks.
- *Digital decision fatigue*: The mental exhaustion and overload resulting from the constant decisions required during online interactions.
- *Facebook depression*: A term introduced by the American Academy of Pediatrics to describe symptoms of depression observed in children and adolescents who spend excessive time on social networks.^6^

The boundary between frequent, non-problematic use and problematic, potentially addictive use of social networks are often subtle. For instance, *FoMO* can lead to habits that gradually evolve **into addiction**, while Nomophobia, initially can be a way to stay connected, can also foster addictive behaviors.

## Mental Health and Social Networks

Social networks promote identity formation and the development of virtual connections, regardless of physical distance. However, they can negatively affect mental health through unwanted feedback, lack of social validation, and negative comparisons. These effects are directly proportional to the frequency of network use and are often linked to sensitivity about personal appearance.^7^

In addition, reliance on technology can impoverish social skills, which are increasingly sacrificed in favor of continuous digital engagement. Shortened attention spans and reduced capacity for information retention have led to individuals being described as *"alone together"*: although connected through technology, they remain isolated in reality.^8^

The symptoms associated with excessive online engagement often resemble those of chemical dependency. The first literature review on social network addiction, published in 2011,^9^ marked a significant milestone in the study of this phenomenon, as research in this field has advanced rapidly since.

Excessive use of social networks by young adults has been associated with lower self-esteem, reduced subjective well-being, and decreased levels of satisfaction with life over time.^10^ Younger generations are particularly vulnerable in developing this addiction, with differing generational perceptions further complicating this issue. Young people often perceive their extensive network use as less problematic than their parents do, viewing it instead as a way of being independent from psychopathological symptoms.

Social networks are frequently used to escape negative emotions, making individuals more vulnerable to anxiety and depression.^11^ A preference for digital socialization also increases the risk of developing social phobia, while cyberbullying exacerbates anxiety and depressive symptoms. Consequently, excessive use of social networks is a strong predictor of depression and poor social connections.

Self-harm, a common symptom of psychiatric disorders in adolescents and young adults, spans a continuum between non-suicidal acts and suicidal ideation. Social networks can expose young people to various methods of self-harm and suicide, creating an alarming sense of helplessness and hopelessness.

## Digital Wellbeing

Neuroplasticity in the brain is shaped by environmental stimuli and how they are processed. The impact is not so much about the technology we use but how we use it: whether for learning, problem solving, or creative expression, we are stimulating positive neuroplasticity, which connects and keeps the neurons active. However, passive, repetitive, or superficial use promotes negative neuroplasticity, weakening these connections.^12^

*JoMO,* the acronym for "Joy of Missing Out," represents the pleasure of intentionally opting out activities or a philosophy of not "getting carried away" by the addictive pull of social networks—contrasting with FoMO.^12^ This more conscious and relaxed mindset encourages satisfaction with personal choices, even when missing out on social events. *JoMO* emphasizes avoiding constant comparison with others, prioritizing personal goals, values, and enjoying one's own company without reliance on external validation.

Psychoeducation plays a vital role in helping individuals make informed choices regarding media consumption. Early interventions often incorporate behavioral approaches to address excessive or addictive use. Among non-pharmacological treatments for Internet addiction, cognitive-behavioral therapy (CBT) shows the strongest evidence of effectiveness.^13^ Unlike substance addiction, treatment for Internet addiction focuses on control rather than abstinence, as the Internet use is deeply integrated into daily life.^14^ Therapy emphasizes on establishing balanced use and increasing awareness of the risks associated with media overuse.

Currently, there is limited evidence supporting pharmacological treatments for social network addiction and other forms of problematic Internet use. Existing research has explored the potential benefits of medications used for other addictions and impulsive-compulsive disorders. Antidepressants and stimulants may alleviate compulsive internet use and online gaming disorders.^15,16^ Additionally, glutamate modulators and opioid receptor antagonists show promise in treating certain behavioral addictions.^17,18^

Future research could explore neuromodulator treatments, such as transcranial magnetic stimulation, which have proven beneficial in other contexts.^19^ However, pharmacological treatment becomes necessary when psychiatric conditions such as depression and anxiety coexist with problematic social network use.
